# Supplementary figures and images for: TaxiBGC: a Taxonomy-Guided Approach for Profiling Experimentally Characterized Microbial Biosynthetic Gene Clusters and Secondary Metabolite Production Potential in Metagenomes
Source: mSystems. 2022 Nov 15;7(6):e00925-22. doi: 10.1128/msystems.00925-22 (PMC9765181; doi:10.1128/msystems.00925-22)

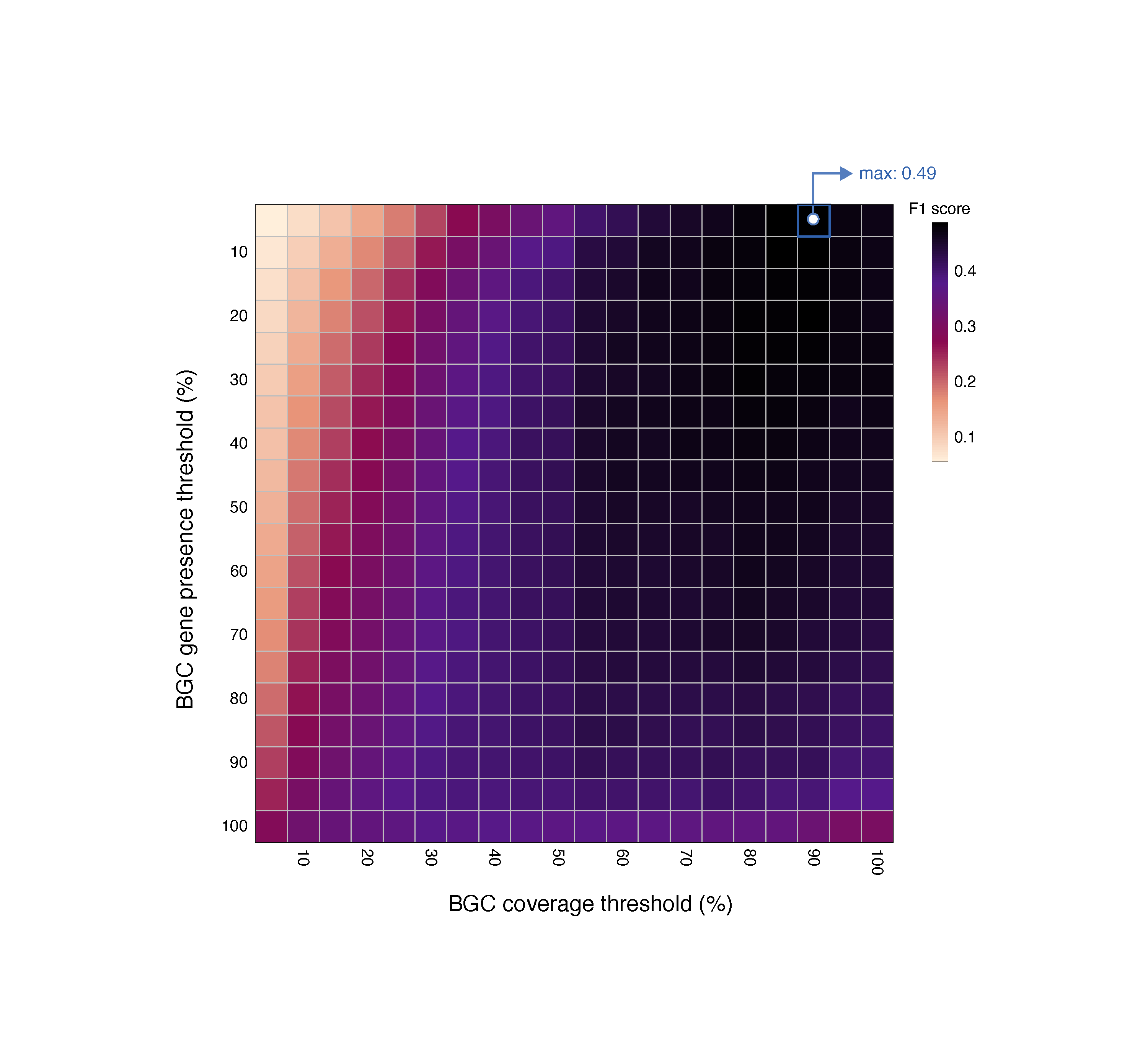

Supplement: FIG S1 [file msystems.00925-22-s0001.tif]

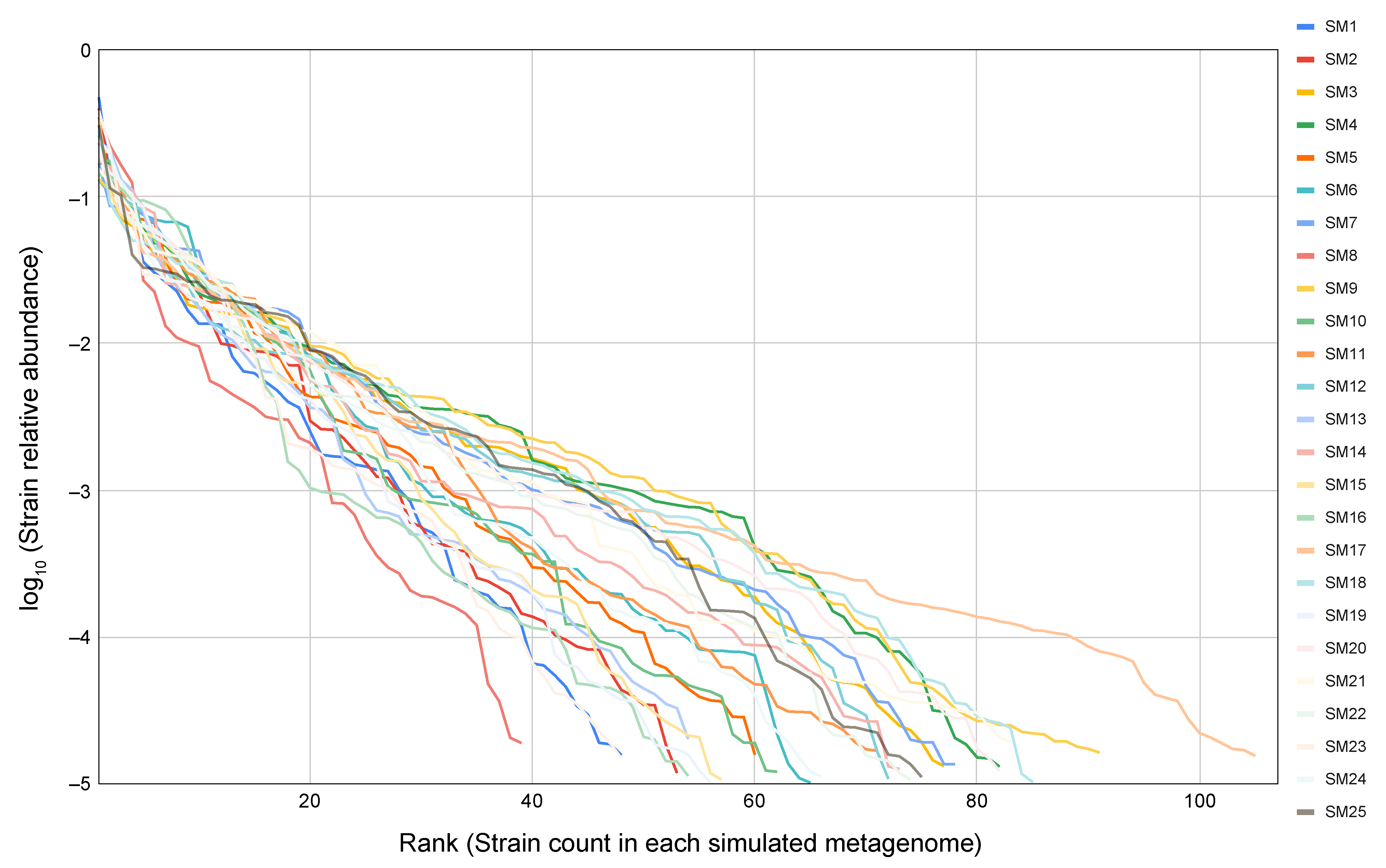

Supplement: FIG S2 [file msystems.00925-22-s0002.tif]
